# New insight in the cross-talk between microglia and schizophrenia: From the perspective of neurodevelopment

**DOI:** 10.3389/fpsyt.2023.1126632

**Published:** 2023-02-16

**Authors:** Jingjing Li, Yu Wang, Xiuxia Yuan, Yulin Kang, Xueqin Song

**Affiliations:** ^1^Department of Psychiatry, The First Affiliated Hospital of Zhengzhou University, Zhengzhou, China; ^2^Henan International Joint Laboratory of Biological Psychiatry, Zhengzhou, China; ^3^Henan Psychiatric Transformation Research Key Laboratory, Zhengzhou University, Zhengzhou, China; ^4^College of First Clinical, Chongqing Medical University, Chongqing, China; ^5^Institute of Environmental Information, Chinese Research Academy of Environmental Sciences, Beijing, China

**Keywords:** schizophrenia, microglia, maternal immune activation, synaptic pruning, inflammation

## Abstract

Characterized by psychotic symptoms, negative symptoms and cognitive deficits, schizophrenia had a catastrophic effect on patients and their families. Multifaceted reliable evidence indicated that schizophrenia is a neurodevelopmental disorder. Microglia, the immune cells in central nervous system, related to many neurodevelopmental diseases. Microglia could affect neuronal survival, neuronal death and synaptic plasticity during neurodevelopment. Anomalous microglia during neurodevelopment may be associated with schizophrenia. Therefore, a hypothesis proposes that the abnormal function of microglia leads to the occurrence of schizophrenia. Nowadays, accumulating experiments between microglia and schizophrenia could afford unparalleled probability to assess this hypothesis. Herein, this review summarizes the latest supporting evidence in order to shed light on the mystery of microglia in schizophrenia.

## Introduction

Schizophrenia is a devastating neurodevelopmental disease characterized by psychotic symptoms, negative symptoms and cognitive deficits (1). Researchers have made mounting advances in understanding of schizophrenia in recent years (2, 3). Hypothesis proposes that abnormal changes during neurodevelopment may cause schizophrenia. What kind of abnormal changes are critical contributors to schizophrenia? The evidence to date indicates the immune cell in central nervous system (CNS)–microglia. With continuous updated views of microglia, it has been discovered that microglia play a significant role in the whole life of neurons-from neurogenesis to the final neuronal apoptosis (4–6). Furthermore, aberrant characteristics of microglia have been found not only in experimental studies but also in clinical schizophrenia patients (7–9). Collectively, it is reasonable to speculate on the role of microglia in schizophrenia.

In this review, we focus on the hypothesis of microglia in schizophrenia. First, we reviewed the relevant clinical evidence, experimental evidence, and findings of antipsychotics in recent years, and then investigated the pros and cons of the hypothesis about microglia in schizophrenia. In the end, we put forward the perspective for future research.

## The link between schizophrenia and microglia in schizophrenia patients

### Changes of schizophrenia-related genes in microglia

With high heritability of more than 50%, schizophrenia genomic architecture shows many hints. Recent large genome-wide association studies demonstrated many genes associated with schizophrenia (2, 3). But the relationship between those genes and microglia remains unclear. By integrating multiple gene approaches, a study found 41 causal genes related to schizophrenia. And among them, ATP2A2, PSMA4, PBRM1, SERPING1, and VRK2 were highly expressed in microglia (10). Ala55Thr, an allelic variant of CX3C chemokine receptor 1 (CX3CR1), has been significantly correlated with schizophrenia while microglia are the only resource expressing CX3CR1 in the brain, point to the significance of these cells and their attendant functions (11). In a nutshell, microglia and schizophrenia have an interesting connection at the genetic level. The microglia-related genes listed here offer an empirically supported resource for understanding of schizophrenia. But in recent largest genome-wide association studies of schizophrenia, researchers found no obvious enrichment of schizophrenia-related genes in microglia or other glia cells (12). But it’s not powerful to refuse microglial function in schizophrenia, more studies are needed.

### Abnormality of microglia in PET imaging of patients with schizophrenia

Recognizing radiolabeled targets-binding compounds, positron emission tomography (PET) can measure metabolic and cellular activities in a non-invasive way. Increased 18 kDa translocator protein (TSPO) in brain represents increasing number of activated microglia and the degree of neuroinflammation (13). By using (R)-[^11^C]PK11195 to combine TSPO and then testing (R)-[^11^C]PK11195 binding potential (BP_*ND*_), no difference was found in the total gray matter and five brain regions including striatum, frontal cortex, thalamus, parietal cortex, and temporal cortex of recent-onset schizophrenia (14). Another PET study, which used same radiolabeled compound, found no difference in the activation state of microglia in anterior cingulate, dorsal frontal, thalamus, medial temporal, orbital frontal and insula either (15). After changing radiolabeled compound into [^11^C]DPA-713, there was still no change in TSPO measured by total volume of distribution (V_*T*_) values in cortical and subcortical brain regions (16). Same result came when using [^18^F]FEPPA PET to observe the dorsolateral prefrontal cortex or the hippocampus of first-episode schizophrenia (17) (Table 1).

**TABLE 1:** Results of PET study of different brain regions and microglial marker radioligands in patients with schizophrenia.

Outcome measure	Brain region	Result	Description of schizophrenia cohort	Radioligand
BP_*ND*_	total gray matter, as well as five gray matter regions of interest (frontal cortex, temporal cortex, parietal cortex, striatum, and thalamus)	no significant differencedierence	19 patients with a recent onset psychotic disorder and 17 age and sex-matched healthy controls	(R)-[11C]PK11195
BP_*ND*_	dorsal frontal (superior and middle frontal cortex), orbital frontal, anterior cingulate, medial temporal (hippocampus and parahippocampus), thala mus and insula	no significant difference	10 individuals at ultra high risk for psychosis, 18 recent-onset schizophrenia patients (illness duration 2 years), 15 chronic schizophre nia patients (illness duration 45 years) and 27 healthy controls matched on age, sex and parental education	11C-(R)-PK11195
V_*T*_	insula, cingulate, parietal, frontal, temporal and occipital, hippocampus, amygdala	no significant change	14 patients with recent onset of schizophrenia (defined as within 5 years of diagnosis) and 16 healthy control subjects	[11C]DPA-713
V_*T*_	the dorsolateral prefrontal cortex, the hippocampus, the medial prefrontal cortex, the temporal cortex, total gray matter, and whole brain	no significant difference	23 untreated patients with psychosis and 20 matched healthy volunteers	[18F]FEPPA
the adjusted distribution volume ratio	total cortical gray matter, temporal, frontal lobe gray matter, regions from the atlas	microglial activity is related to the altered cortical volume seen in schizophrenia	14 subjects meeting ultra high risk criteria,14 subjects with schizophrenia, 22 healthy control subjects	[11C]PBR28
BP_*ND*_	anterior cingulate cortex, prefrontal cortex, orbitofrontal cortex, parietal cortex, putamen, thalamus, and brainstem	aging is associated with higher TSPO but a diagnosis of schizophrenia is not	20 recent onset patients, 21 established patients, 21 age- and sex-matched controls	[11C](R)-PK11195
[11C]PBR28 binding ratio	total graey matter, temporal, frontal lobe graey matter	elevated microglial activity	14 subjects meeting ultra high risk criteria,14 subjects with schizophrenia, 28 age (± 5 years) matched control subjects	[11C]PBR28
V_*T*_	gray matter, white matter, frontal cortex, temporal cortex and hippocampus	reduced [11C]PBR28 V_*T*_	16 first-episode drug-naive psychotic patients,16 control subjects	[11C]PBR28
BP_*ND*_	dorsolateral prefrontal, ventrolateral, orbitofrontal, anterior cingulate, parietal	not increased in antipsychotic-free patients and elevated in medicated patients	16 patients with a diagnosis of schizophrenia, 16 healthy volunteers	[11C](R)-PK11195

BP_*ND*_, binding potential; V_*T*_, total volume of distribution.

However, when changing into other radioligands, things become different. In [^11^C]-PBR28 PET studies, TSPO increased in both schizophrenia and high-risk groups. Besides, TSPO was negatively correlated with cortical volume, while cortical volume loss was observed in schizophrenia (18–20). But it’s hard to conclude that different radioligands affect the results of TSPO. Antipsychotic medications may have the ability to change microglia activation too. A study showed TSPO decreased in the first-episode untreated schizophrenia compared with medicated groups. Another study found no significant difference in TSPO between untreated patients and control group, but statistical increase in TSPO after medication treatment (21–23). The limited samples may be reason too. A meta-analysis suggested that TSPO in frontal cortex, hippocampus and temporal cortex were reduced in schizophrenia (24). Lower non-displaceable binding in schizophrenia may also interfere with the results. Therefore, Marques et al. (25) used XBD173, a TSPO ligand, to block [^11^C]-PBR28, but found no significant difference in non-displaceable binding between schizophrenia and healthy controls. However, not only microglia, astroglia and endothelial cells express TSPO as well (13). To assess the role of microglia in schizophrenia need more specific microglial targets.

### Post-mortem results in which microglia involved of schizophrenia

Neuroinflammation and immune environment play an important role in schizophrenia. Fc receptors can combine with antigen-antibody complex and transfer information into cells. In a post-mortem study, microglial Fcγ receptors (CD64, CD64/HLA-DR) was elevated in the dorsal prefrontal cortex of schizophrenia. Higher ionized calcium binding adapter molecule 1 (IBA1) and CD64/HLA-DR rate appeared in patients with psychotic symptoms while in death (7). Changes in microglial Fcγ receptors manifest that the systemic immune system interacts with microglia, thus altering brain immune environment. To decline the effect of confounders–brain cytokines, the high brain cytokines subtypes of both schizophrenia and controls were compared. Subtypes of schizophrenia showed weaker nuclear factor kappa B (NF-κB) activation which related to HIVEP2 mRNA reduction. To confirm this, HIVEP2 knock-out mice decreased microglial NF-κB transcripts (26). Though post-mortem studies have limitations, we still have a glimpse of unusual microglial action in schizophrenia.

### Changes in body fluids in schizophrenia patients related to microglia

To further explore how peripheral compounds influence microglia, we focus on the changes in body fluids then. The peripheral blood serum extracted from drug-naive first-onset schizophrenia patients can activate microglia *in vitro*, which can be blocked by proinflammatory activation inhibitors (8). Furthermore, the receptor for advanced glycation end-products (RAGE) increased in the serum of patients with schizophrenia, and higher RAGE was related to low prefrontal GABA levels (27). Changes have also occurred in the leukocytes, CX3CR1 was down-regulated in peripheral blood monocyte (28). TERM2 mRNA, which related to microglia activation, was elevated in leukocytes and then declined after treatment of clozapine (29). Another study turned monocyte from blood into induced microglia-like phenotype (iMG) which had high heterogeneity regardless of group. But iMG subtype expressing ApoE, Ccr2, CD18, CD44, and CD95, as well as iMG subtype expressing IRF8, P2Y12, CX3CR1, and HLA-DR were main subtypes in schizophrenia. Moreover, iMG induced from schizophrenia responded more strongly to lipopolysaccharide (LPS) and increased TNF-α secretion (30). In a study of cerebrospinal fluid extracted from twins with and without psychotic disorders, soluble cluster of differentiation 14 (sCD14), a marker of microglia, was exaggerated in the one with schizophrenia or bipolar disorder (31). The evidence showed that unknown changes in the peripheral blood and cerebrospinal fluid of schizophrenia were somehow related to microglia, but the specific mechanism needs further research.

In clinical researches, microglia and schizophrenia have shown a moderate link. Several risk genes were related to microglia, although it’s a small part of hundreds of risk genes of schizophrenia. One drawback of PET studies is that TSPO is not specific for microglia and, therefore, PET studies need more unique probes for microglia. Post-mortem results and body-fluid changes in schizophrenia have led to the belief that abnormal microglia indeed appear in schizophrenia.

## The link between schizophrenia and microglia in animal models

Complementary to human studies, animal models provide a legible opportunity to understand underlying mechanisms between schizophrenia and microglia.

### Altered microglial function in MIA models

Maternal immune activation (MIA) is a wide-used animal model to explore molecular mechanism of schizophrenia. An epidemiologic study in 1964 has indicated that infection during gestation enhances the incidence rate of schizophrenia (32). Many agents such as influenza, polyinosinic:polycytidylic acid [Poly (I:C)], LPS and IL-6 (33) are used to induce maternal immune response. Generally, the immunogen is injected into maternal body during middle or late stage of pregnancy. Then researchers study the offspring in different periods (Table 2).

**TABLE 2:** Changes in microglia in the MIA model.

MIA model	Immunological manipulation	Stimulation time	Observation time	Brain area	Result
CX3CR1-eGFP transgenic mice	Poly (I:C) (20 mg/kg) or vehicle (saline)	embryonic day 11.5 (single injection)/embryonic day 11.5 and 15.5 (double injection)	embryonic day 17.5	fetal cortex, hippocampus	no significant differences in fetal microglial cell density or immunohistochemically determined activation level
Long-Evans rats	Poly (I:C) (4 mg/kg) or saline	gestational day 15	postnatal day 7/postnatal day 21/postnatal day 35/postnatal day 90	all brain regions (medial prelimbic cortex, frontal association cortex, amygdala, primary auditory cortex)	no obvious changes in microglia across all regions
CD-1 mice	Poly (I:C) (20 mg/kg) or 0.9% vehicle	gestational day 9	postnatal day 170	neocortex, hippocampus	enrichment-induced enhancement in microglia prevented by MIA
C57BL6/N mice	Poly (I:C) (5 mg/kg) or 0.9% vehicle	gestational day 17	postnatal day 120	midbrain	unchanged microglial cell numbers in the midbrain
C57BL/6J mice	Poly (I:C) (20 mg/kg) or saline daily for 3 days	embryonic day (11–13)/embryonic day (15–17)	not mentioned	frontal cortex	no differences in NF-κB-related mRNA levels
BALB/c mice	Poly (I:C) potassium salt (20 mg/kg)or 0.9% saline	gestational day 9.5	postnatal day 30	hippocampus, corpus callosum, striatum, cortex, fimbria, ventricle	overactivation of proinflammatory responses in microglia
CX3CR1-EGFP transgenic mice	Poly (I:C) (10 mg/kg) or saline	gestational day 12/gestational day 15	gestational day 18/postnatal day 10	brain	altered motility and cytokine-release level of microglia
C57BL6/N mice	Poly (I:C) (5 mg/kg) or vehicle	gestational day 9	postnatal day 21/postnatal day 40/postnatal day 90	dorsal cornu amonis, dentate gyrus	unchanged hippocampal microglia density or activation statuses
C57BL/6 mice	Poly (I:C) (5 mg/kg) or 0.9% saline	gestational day 15	not mentioned	hippocampus	altered transcriptome signature of hippocampal microglial cells
C57Bl/6 mice	Poly (I:C) potassium salt (5 mg/kg)or vehicle	embryonic day 9.5	postnatal day61–81/postnatal day80–90	hippocampus	increased CD68 levels in IBA1 + microglia of female but not male offspring
BALB/c mice	Poly (I:C) (20 mg/kg) or 0.9% sterile sodium chloride	gestational day 9	postnatal day 30/postnatal day 100	brain	post pubertal deficits in sensory gating preceded by a pro-inflammatory activation pattern of microglia during puberty
New Zealand white rabbits	LPS (8,000EU) or intravenous fluids	gestational day 28	gestational day 29	fetal brain, placenta	shunted tryptophan metabolism away from the serotonin to the kynurenine pathway by maternal inflammation
Wistar rats	LPS (2 mg/kg)/Poly I:C (4 mg/kg)	gestational day 7 and every second day until delivery/gestational day 15	postnatal day 7/postnatal day 93	hippocampus, frontal cortex	altered developmental trajectories in neuron-microglia communication
Wistar rats	Poly (I:C) (10 mg/kg) or saline	gestational day 15	gestational day 21/postnatal day 21	hippocampus	increased amoeboid cells in the hippocampus of the male offspring

In rodent MIA model, the performance of microglia is bewildering. The density and morphology of microglia in embryonic and adult stages remained unchanged. The nuclear NF-κB-related mRNA levels in the offspring did not change either (34–36). Besides, microglia showed unchanged density in frontal association cortex, amygdala, medial prelimbic cortex, and primary auditory cortex of offspring (37). But microglial density has no capacity to show full profile of microglia. More details of microglia are needed. Buschert et al. (9) found MIA could prevent increased microglia which caused by environmental enrichment, and those microglia possessed a neurotrophic phenotype. Another research showed that microglia had abnormal motility and cytokine-release ability. And the microglial density increased with maternal LPS treatment (38–40). Furthermore, MIA could affect mRNA level, protein level and membrane surface protein level of CX3CL1-CX3CR1 and CD200-CD200R signaling pathways in hippocampus and frontal cortex of the offspring (41). Moreover, MIA can activate Toll-like receptor (TLR) and inflammasome pathways (42). Taken together, microglial characteristics were altered during neurodevelopment in MIA model. This may imply abnormal microglia is related to schizophrenia.

Variations in the hippocampus have been extensively studied. After isolating and analyzing microglia from hippocampus of adult offspring with abnormal behaviors, Mattei et al. found the transcription of microglia was altered–genes related to inflammation expression, cell migration and phagocytosis have been down-regulated, while those related to neuron-plasticity regulation have been up-regulated. Besides, increased microglial density and amoeboid cell numbers (represent activated microglia) were found in the hippocampus of male offspring (43, 44). However, another study showed that in the hippocampus of juvenile (postnatal day 21), adolescent (postnatal day 40) and adult (postnatal day 90) offspring, no increase in IBA1 + or CD68 + microglia, and no morphological changes that implies activation were found (45). Conflicting results on microglial density and morphology imply that perhaps some other factors affect microglia. A great effort is required to reveal the mechanism of microglia in MIA model.

In rodent models, microglial changes had gender difference. Compared with males, MIA can increase the microglial CD68 expression of female offspring in the hippocampal dentate gyrus (46). Besides, the female offspring (100 day-old) with sensory gate defects appeared strong M1-type microglia during adolescence (47). The results showed sex differences while microglia in the brain responded to MIA.

In rabbit MIA model, more detailed mechanism has been explained. After intrauterine injection of LPS in rabbits at gestational day 28, indoleamine 2,3-dioxygenase (IDO) was found to increase in placenta and fetal brain. The increased IDO was associated with activated microglia. Besides, IDO can activate the kynurenine (KYN) pathway to convert tryptophan to KYN, while microglia was capable of converting KYN to quinolinic acid. Quinolinic acid could increase oxidative stress and cause excitotoxicity, which related to some neuropsychiatric diseases including schizophrenia (48). The abnormal microglia in rabbit MIA model could lead to neuroinflammation, oxidative stress, and severe damage.

### The role of microglia in toxoplasma gondii-related schizophrenia

Toxoplasma gondii is an intracellular protozoan parasite with a significant tendency to infect the CNS. It has been found to be related to the increased incidence of many mental diseases. A host of behavioral studies have proved that Toxoplasma gondii can cause impaired athletic ability, spatial learning and dysmnesia. Given that schizophrenia may be a neurodevelopmental disorder, the age of first exposure to Toxoplasma gondii is also an important factor in the formation of different behaviors and neurobiological abnormalities. The expression of IBA1 in the brains of infected mice was related to age. The underlying mechanism may be to increase the N-methyl-D-aspartate receptor (NMDAR) subunit GLUN2A or complement protein C1q of autoantibodies (49). Above evidence further strengthen the view that microglia play an anti-inflammatory role in schizophrenia. There is another more convincing study. Glutamate decarboxylase 67 (GAD67) catalyzes the inhibition of GABA synthesis in synapses. The persistent infection of Toxoplasma can change the distribution of GAD67, resulting in contact between microglia and neurons, loss of peripheral inhibitory synapses, and resulting in myeloid-derived cells wrapping neuronal cells, thereby increasing risks of schizophrenia (50).

Regardless, many studies regard toxoplasma exposure as a risk factor for a suite of other psychiatric disorders, including schizophrenia. The effects of Toxoplasma infection on neuroinflammation are only in its infancy. Toxoplasma gondii can cause synaptic pruning and complement changes in microglia to a large extent by affecting GABA and glutamate, striking us that we can relieve or cure the symptoms of mental disorders by reducing the parasite’s antigen utilization.

In animal models of schizophrenia, the detailed mechanisms are clarified. MIA is a well-established model to explore schizophrenia, especially in neurodevelopment. Results indicated that inner changes in microglia happened during neurodevelopment. Toxoplasma gondii infection also demonstrates this.

## Blunter internal inflammatory pathway of microglia under the treatment of antipsychotic medications

**FIGURE 1:**
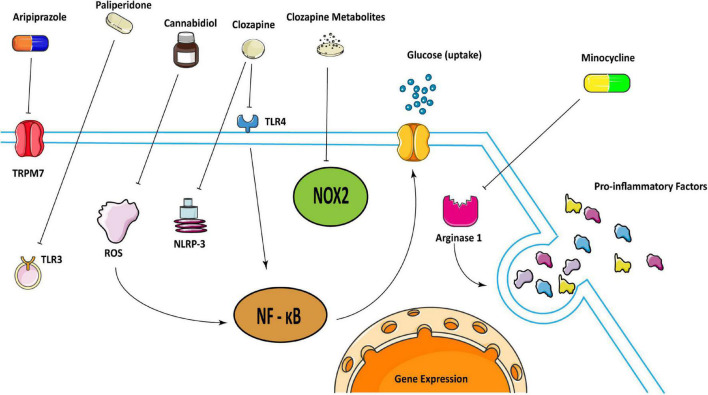
Schematic illustration representing how some antipsychotics may treat schizophrenia in regulating microglia. Clozapine can attenuate inflammatory responses by inhibiting the activation of TLR 4/NF-κB mediated by Ca^2^ + /CaM/Akt signal as well as inhibit the activation of NLRP-3 inflammasome. And clozapine metabolites can inhibit NOX2 to inactivate microglia. Cannabidiol may inhibit microglial glucose-uptake by inhibiting ROS/NF-κB-dependent signal transduction to exert anti-inflammatory effects. Aripiprazole can inhibit microglia activation through TRPM7, and paliperidone can achieve the same result through TLR3. Besides, minocycline could reduce pro-inflammatory factors through arginase 1.

Antipsychotic medications can effectively control the mental symptoms of schizophrenia. The first-generation antipsychotic medications (typical antipsychotic medications) mainly act on the central dopamine D2 receptor and are effective in the treatment of positive symptoms of schizophrenia. The second-generation antipsychotics (atypical antipsychotics) are gradually becoming wide-used because of their greater efficacy in treating negative symptoms of schizophrenia and fewer side effects (51–53). Ample studies have found that antipsychotic medications work on neuroinflammation caused by microglia (54) (Figure 1).

### The changed function of microglia under clozapine

A study showed that clozapine inhibited the function of microglia by inhibiting the activation of TLR 4/NF-κB mediated by Ca^2^ + /CaM/Akt signal in microglia, and attenuating microglial inflammatory responses (55). Besides, clozapine could reduce the expression of chemokines by targeting primary microglia in an animal model (56, 57). Another study showed that clozapine can inhibit the activation of NLRP-3 inflammasome in primary microglia induced by Poly (I:C) to inhibit the expression of pro-inflammatory factors (58). More than that, clozapine metabolites clozapine N-oxide and N-desmethylclozapine could also inhibit NADPH oxidase 2 (NOX2) to inactivate microglia and protect dopaminergic neurons (59). Taken together, Clozapine can inhibit the function of microglia through a variety of internal mechanisms that are critical in neuroinflammation.

### The changed function of microglia under paliperidone and aripiprazole

Paliperidone and aripiprazole are atypical antipsychotic medications. The former can improve the cognitive function and overall social function of patients but its exact mechanism is still unclear. In maternal immunity model, paliperidone could inhibit the TLR 3 signaling pathway in young adult mice exposed to maternal immune challenges before delivery, and stimulate the polarization of microglia to the anti-inflammatory M2 type (60). Aripiprazole inhibited Poly (I:C)-induced microglia activation through transient receptor potential in melastatin 7 (TRPM7) (61).

### The changed function of microglia under cannabidiol

Cannabidiol (CBD) also has a potential therapeutic effect on schizophrenia which resembles atypical antipsychotics (62–64). Unlike clozapine, CBD could activate 5-HT1A receptors in rodent models (65). And it might also inhibit the uptake of glucose by microglia by inhibiting ROS/NF-κB-dependent signal transduction to exert anti-inflammatory effects (66). In addition, most of the genes affected by CBD are distributed in microglia, mediating synaptogenesis, and they can improve cognitive impairment in schizophrenia and reverse the cortical transcription changes (67). Another study also verified that CBD may have antipsychotic effects for it can improve social interaction and spatial learning and memory disorders in animal models of schizophrenia caused by MK-801, which is also known as dizocilpine, increase the percentage of IBA1 + for the reactive phenotype microglia marker in the mPFC and dorsal hippocampus, but does not change the number of IBA1 + cells (64).

### The changed function of microglia under minocycline

Minocycline is a semi-synthetic tetracycline with good diffusion in the brain. It has been shown to be effective in treating neurodegenerative diseases in animal models (68). But its ability to treat early schizophrenia is still slim (69, 70). Perhaps it is due to the fact that the inflammation is not obvious at this early period that it is not necessary to use it at this time. However, when minocycline is combined with risperidone, it can improve the negative symptoms of schizophrenia, which may be related to the reduction of pro-inflammatory cytokines by inhibiting microglia (71, 72). Minocycline inhibited the activation of microglia, changed its transcriptome and phagocytic function, increased the density and somatic cell size of rat hippocampus and nucleus accumbens, and reduced behavioral defects in schizophrenia (73, 74). It could also increase C3 and C1q mRNA in a maternal immune model (75, 76). Moreover, presymptomatic minocycline treatment in rodents may prevent behavioral abnormalities in neuropsychiatric disorders including schizophrenia (77). It may mediate the selective activation of microglia through arginase 1 (Arg1) (78). Another study showed that minocycline reduced microglia-mediated synaptic uptake *in vitro* (79). Based on its neuroprotective and anti-inflammatory effects, minocycline was also recommended for the treatment of neuropsychiatric symptoms of coronavirus COVID-19 (80).

Hence, antipsychotic medications could affect microglia, which may provide proof of microglial involvement in schizophrenia.

## The hypothesis of microglia and schizophrenia

Large evidence has unraveled that schizophrenia is a neurodevelopmental disease. Companied with intricate and exquisite function of microglia in CNS, a hypothesis believes that during the process of neurodevelopment, the peculiarities of microglia have changed, contributing to brain dysfunction, which may be the basis for the pathogenesis of some CNS diseases including schizophrenia. Afterward, more attention was paid to the abnormal characteristics of microglia in schizophrenia, thus enriching this hypothesis. Nowadays, the hypothesis verged to be sophisticated. It’s suggested that the interaction of glial cells in the CNS can lead to the development of schizophrenia (81). However, microglia still play a key role in the developed hypothesis. Moreover, some believed that abnormalities in microglia exist in not only schizophrenia but also many other neurodevelopmental diseases (82).

To the best of our knowledge, except for the genetic link between microglia and schizophrenia, abnormal microglia were also observed in brain imaging, post-mortem and body fluid studies in patients with schizophrenia. This abnormality is special for schizophrenia. It is therewith wondering whether the abnormalities of these microglia may precede the onset of schizophrenia or be the consequence of schizophrenia. Then, in animal models, the abnormal microglia occurred during neurodevelopment. In addition, the antipsychotic agents have altered microglia which may be one of the underlying mechanisms for drug therapy. To date, it still remains unclear what the exact role of microglia plays in schizophrenia.

To date, on the account of the limitation of animal models and the exquisite sensitivity of microglia, the studies on internal molecule mechanisms of microglia in schizophrenia are finite, which may to some extent weaken this hypothesis. But it is worthy of affirmation that the study of the changes in microglia is of great significance.

## Perspective and conclusion

It should be noted that there may be different changes in microglia in different brain regions. Herein, drawing a complete single-cell sequencing map of the changes in the characteristics of microglia in each brain region will provide a lot of information for the study of schizophrenia. At present, patients with schizophrenia are mainly classified based on clinical manifestations, onset time, etc. Although this classification is helpful for treatment, it will be more conducive to the treatment of schizophrenia with biological classification. It’s proposed that schizophrenia can be classified according to immunophenotype and inflammation of cytokines. Since the immune and inflammatory environment of the brain are dependent on microglia, further research on microglia may provide new ideas for classification. In addition, there is a study showing that the internal pathways of microglia in patients with high-inflammatory schizophrenia are not consistent with high-inflammatory controls (26). This gives us a hint that microglia play a role in schizophrenia not only in neuroinflammation, but also in other unknown parts. To date, it has been found that Synaptotagmin-11 can prevent microglia, and have the clinical use potential (83). More researches in this field will help discover innovative treatment options. Traditionally, the mechanism of antipsychotic drugs is mainly through blocking receptors including serotonin receptors. Currently, the mechanism studies have found that these antipsychotic drugs also affect microglia. How important is the effect of antipsychotic drugs on microglia in the treatment of schizophrenia? It is worth considering. If it is significant enough, the treatment plan for schizophrenia may start a new chapter.

In summary, we found that the hypothesis about the role of microglia in schizophrenia is worthy of attention, and further research may bring new perspectives and ideas to this hypothesis.

## Author contributions

JL and YW designed the review outline and wrote the manuscript. XS, XY, and YK critically reviewed and revised the manuscript. All authors approved the final manuscript.
